# Lower bone mineral density in older female endurance skiers – a cross-sectional, observational study

**DOI:** 10.1186/s11556-018-0202-1

**Published:** 2018-11-17

**Authors:** Anne K. Gulsvik, Marius Myrstad, Ida Wilson Landgraff, Nina Emaus, Anette Hylen Ranhoff

**Affiliations:** 1 Department of Internal medicine, Diakonhjemmet hospital, Pb 23 Vinderen, 0319 Oslo, Oslo, Norway; 2 Department of Medical Research, Bærum Hospital, Vestre Viken Trust, Bærum, Norway; 3 Department of Orthopaedic Surgery, Bærum Hospital, Vestre Viken Trust, Bærum, Norway; 4 Department of Health and Care Sciences, Faculty of Health Sciences, UiT The Arctic University of Norway, Tromsø, Norway; 5 Department of Clincal Science, University of Bergen, Diakonhjemmet Hospital, and National Institute of Public Health, Oslo, Norway

**Keywords:** Aging, Bone mineral density, Exercise, Fractures, DXA, Osteoporosis

## Abstract

**Background:**

Physical activity (PA) is generally beneficial for bone health, but the effect of high levels of PA over many years, in older women, is unknown.

**Methods:**

T-score from Dual-energy X-ray absorptiometry (DXA), and self-reported baseline characteristics were recorded for 24 female, cross-country-skiing-competitors, aged 68–76 years, from the Birkebeiner Ageing Study. Data from 647 women in the same age range from the Tromso-6 population study, with recorded DXA findings, were used for comparison.

**Results:**

The athletes reported a median(range) of 9(1–34) participations in the 54 km, yearly ski-race, indicating long-term PA. They also reported more moderate and high levels of PA than women in the general population (52% vs. 12 and 30% vs. 0%, respectively). The athletes had lower body mass index (BMI) than the controls (mean BMI 21.7 vs 26.9 kg/m^2^, *p* < 0.001). As many as 22/24(92%) of the athletes and 477/647(74%) of the controls had a low bone mineral density (BMD) (T-score < − 1), p 0.048, Pearson chi square test. Odds ratio (OR) of low BMD was 3.9 in athletes vs. controls (p 0.048, logistic regression), but adjusting for BMI largely diminished the effect estimate, which was no longer statistically significant (aOR 1.81, p 0.432). The proportion of self-reported fractures was the same in the two groups.

**Conclusions:**

This pilot study suggests that long-term, high levels of PA are associated with low bone mineral density in older women, and the finding might be due to differences in BMI. Despite the lower bone mineral density the athletes did not report more fractures.

## Background

Osteoporosis, defined by low bone mineral density (T-score equal or less than 2,5), is associated with increased bone fragility and susceptibility to fractures. Bone mineral density decreases with age [1]. It is mainly elderly individuals who suffer from osteoporotic fractures, and fractures in the elderly could be associated with serious consequences like reduced mobility and independence, and increased risk of institutionalization and death [2–4]. Thus, it is crucial to improve knowledge on modifiable risk factors for osteoporosis. Modifiable risk factors for osteoporosis and fragility fractures include physical inactivity, low body mass index (BMI), smoking and falls [5, 6], where physical inactivity is one of the most important. In general populations, higher levels of physical activity are positively associated with bone mineral density and associated with lower risk of fractures. There are numerous of studies that have looked at impact of physical activity on bone health in young and middle-aged athletes [7, 8], but few have investigated the effects of perpetual endurance exercise in elderly athletes. Physical activity habits in the population are changing, with an increasing part of the middle aged and old female population participating in recreational sports and competitions like marathon running and cross-country ski races over the past few decades [9, 10]. Whether such activities improve bone health by preventing osteoporosis and fragility fractures in old age needs to be explored further. A few studies have looked at bone strength in elderly athletes and suggest that high impact exercise is effective in maintaining bone strength throughout life [11, 12]. One study, found that cortical bone mineral content was inversely related to running distance and geometrical tibial measures were positively related to running speed [13]. Our study is the only study known to us, which provides data on bone health in long-term, older, female athletes.

### Aim of the study

The aim of this pilot study was to compare the prevalence of low bone mineral density and fractures in older female athletes with those of women in the general population.

## Methods

### Study design and participants

The Birkebeiner Ageing Study is an observational, longitudinal study of men and women aged 65 years and above who competed in the annual Norwegian Birkebeiner cross-country ski race [14]. The Birkebeiner Ageing Study purpose is to investigate the association between long-term exercise and health in advanced age. With a course of 54 km and a total elevation change of 1000 m, the Birkebeiner race is among the world’s most challenging ski races and we presume that repeated participations require regular exercise over a long time. Competitors were invited to participate if they were 65 years of age or older when they completed the race in 2009, 2010 or 2011. This sub-study on bone health included only the women from the Birkebeiner Ageing Study. Their measured bone health data (T-score and BMD) and self-reports on fracture history were compared to a less physically active control group. The control group consisted of women aged 68–76 years who had participated in the Tromsø-6 study. This is a population-based study of people living at home, and with largely the same ethnicity as the Birkebeiner Ageing Study group, and took place from 2007 to 2008 [15, 16].

Potentially confounding variables, including measured body weight, height, and BMI as well as self-reports on several other variables were scrutinized and tested for adjustments.

### Procedure

Of the 55 women invited at baseline in 2009, 46 (84%) answered the initial postal questionnaire. Additionally 14 female athletes were included in 2011. Of the total 60 initial respondents, 46 completed a follow-up questionnaire in 2014 (further referred to as the athletes). By then they were 68–76 years old.

**Fig. 1:**
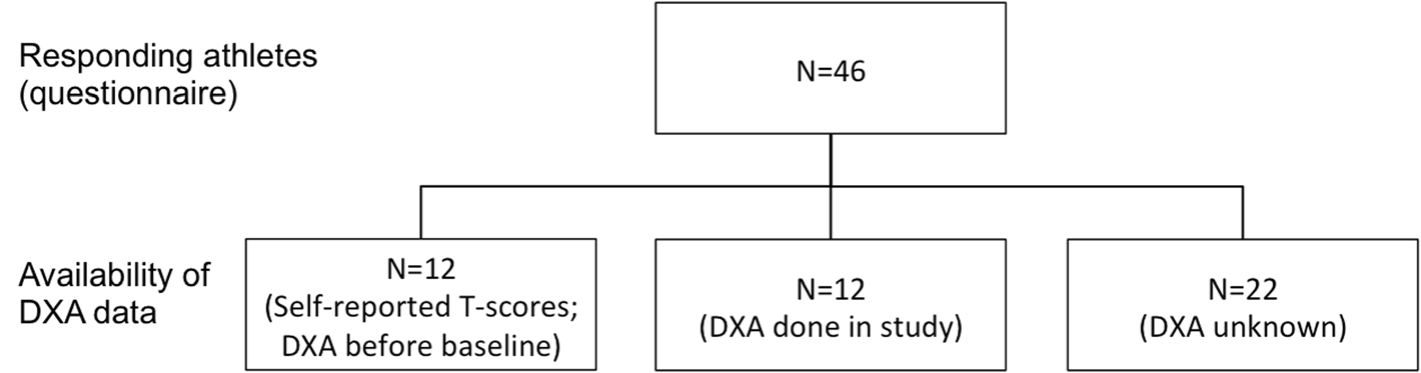
Availability of DXA data from responding athletes

At follow-up in 2014, 12 participants reported already known T-scores measured by DXA, 10 of whom also reported their specific BMD measurements. All provided a copy of a physician report. In addition, 12 athletes, living close to the two largest cities in Norway (Bergen and Oslo), completed Dual-energy X-ray absorptiometry (DXA) of hip and spine as part of the study (September 2014 to February 2015). In total, 24 (12 + 12) T-scores and 22 (10 + 12) BMD measurements from the hip were available for further analyses (Fig. 1). Baseline data for the 22 women without DXA were used to study generalizability. In total, 647 (85%) of the women from the Tromsø-6 study had available DXA data (85%) and were included in this analysis.

### Questionnaires

The questionnaires used in the Birkebeiner Ageing Study and in the Tromso-6 study are available on https://www.haraldsplass.no/helsefaglig/forskningsgruppe-for-geriatri-og-demens/birkebeiner-aldringsstudien-bias and http://www.tromsostudy.com. They contained largely identical questions, allowing direct comparison between the two study populations. Physical activity during the past 12 months was assessed with a validated questionnaire previously used in large population-based studies [17]. On a four-level scale, moderate physical activity was defined as “light sports or heavy gardening for at least four hours per week”. The highest level of physical activity was defined as “regular, hard exercise or competitive sports several times per week”. Previous history of physical activity level was not reported for the controls, but the athletes reported total number of times they had participated in the Birkebeiner ski race. BMI was calculated based on objectively measured weight and height.

### Dual-energy X-ray absorptiometry (DXA)

As part of this study a DXA scan was offered and performed in 12 eligible athletes. Ten of these were measured with Hologic Discovery at Diakonhjemmet hospital, and 2 with GE Lunar at Haukeland University Hospital in Bergen. The DXA scans in the Tromso controls were performed with GE Lunar Prodigy. The location and equipment for the 12 DXA scans performed before the study baseline (based on reports) varied from the city of Trondheim in the north to Fredrikstad in the south (7 Lunar, 2 Hologic, 1 Norland device and 2 unknown). BMD (g/cm^2^) from the femoral neck was used for comparison. T-scores for the Birkebeiner athletes were automatically calculated and provided from the DXA measuring devices. For the Tromso controls the T-scores were calculated using the Lunar reference and with the formula (BMD_measured_-BMD_reference_)/SD_reference_ (BMD_reference_ = 0.98, SD_reference_ = 0.12) [18]. T-scores were used to diagnose osteoporosis and osteopenia according to the definition from the WHO; T-score ≤ − 2.5 was classified as osteoporosis, T-score < − 1.0 and > − 2.5 as osteopenia, T-score ≥ − 1 as normal bone mineral density, hence T-score < − 1 was named low bone mineral density [17]. Standardized BMD (sBMD) was calculated to compare BMD findings across the different DXA devices used [19]. We used the formula sBMD =1000(a + b*BMD). a = 0.019, − 0-023, and 0.006, and b = 1.087, 0.939, and 0.985 when BMD was measured with a Hologic, Lunar and Norland device respectively.

### Statistics

Chi-square, *t-*tests, and ANOVA were used when appropriate. Logistic regression was performed to study the association between a dicothomized BMD (normal vs low) and a dichotomized physical activity level (athletes vs controls). Adjustments for multiple variables were carried out in the interim analyzes, leaving age and BMI as the main factors of interest. All analyses were computed with IBM SPSS (version 25) software.

## Results

The athletes reported substantially higher levels of physical activity during leisure time the past 12 months than the control group. The highest level of physical activity was reported by 7/23 (30%) of the athletes and for none in control group, while 12/23 (52%) and 60/522(12%) reported moderate exercise levels respectively. The athletes had participated in the Birkebeiner ski race for several years (median 9, range 1–34) and are therefore considered long-term athletes.

The main characteristics and findings are further presented in Table 1.

**Table 1:** Characteristics and main findings for the older, female athletes with available T-scores (Birkebeiner Ageing Study), and for women from the Tromso-6 study, aged 68 to 76 years

	Birkebeiner ageing study *N* = 24	Tromso-6 *N* = 647	*P*-values^a^
Age (years) Mean (SD)	71.1(1.5)	71.5(2.6)	0.416
College/university education n/N(%)	15/24(63)	101/638(16)	< 0.001
Body height (m) Mean (SD)	1.65(0.05)	1.60(0.06)	< 0.001
Body mass (kg) Mean (SD)	59.3(4.8)	69.1(11.7)	< 0.001
BMI kg/m^2^ Mean(SD)	21.7(1.6)	26.9(4.5)	< 0.001
Hormone supplement affecting menopause (ever) n/N(%)	11/22(50)	33/537 (6)	< 0.001
Cod liver oil intake daily (current) n/N(%)	16/24(67)	197/519(35)	0.016
Vitamins intake daily (current) n/N (%)	14/22(64)	134/506(26)	< 0.001
Osteoporosis medication (ever) n/N (%)	5/24(21)	80/619(13)	0.262
Self-reported hip fracture n/N (%)	0/24(0)	10/580(2)	0.517
Self-reported wrist fracture n/N (%)	4/24(17)	153/605(25)	0.338
Standardized BMD (g/cm^2^), Mean (SD)	0.710(0.079)	0.730(0.108)	0.404
Femoral neck T-score Mean (SD)	−1.780(0.808)	−1.485(0.958)	0.137
Osteoporosis (T-score ≤ −2.5), n/N (%)	4/24(17)	86/647(13)	0.048 ^b^
Osteopenia (−1 > T-score > −2.5), n/N (%)	18/24(75)	391/647(60)	
Normal BMD (T-score ≥ −1), n/N (%)	2/24(8)	170/647(26)	

*BIAS* Birkebeiner Ageing Study

*SD* standard deviation

*BMI* Body Mass Index

^a^*t*-test, ANOVA, and Pearson chi square test used when appropriate;

^b^chi square comparing normal BMD to low BMD (osteoporosis+osteopenia)

Being an athlete was associated with a greater risk of low BMD in the femoral neck (T-score < − 1; (osteoporosis+osteopenia) OR 3.9 (p 0.048, logistic regression). The effect estimate was not affected by adjustments for age, but the association could not be confirmed after additional adjustments for BMI, adjusted OR (aOR) 1.81 (*p* = 0.432) or after adjustments for body weight, aOR 1.92 (*p* = 0.385). The maximum BMI in the athlete group was 23.88 kg/m^2^. In a subgroup analysis, including only controls with a BMI of 23.88 kg/m^2^ and below (*N* = 173), being an athlete was no longer associated with a low BMD, OR 1.61 (*p* = 0.538).

The prevalence of self-reported (ever) fractures in hip and wrist (combined) did not differ between the athletes and Tromso-6 controls (*p* = 0.299, Pearson chi square). This finding remained unchanged also after including fractures from the 22 athletes without DXA reports (11/46(24%) vs 163/605 (27%), p 0.885, for the athletes and Tromso-6 controls respectively).

Age at menarche was the same in the athletes and the control group (data not shown), but other information regarding previous menstruation cycles or menopausal age was not recorded. The median number of childbirths seemed fewer among the athletes (2 vs 3), although not statistically significantly different. The two cohorts did not differ significantly in prevalence of diabetes, hypothyroidism, and history of ever-smoking, and alcohol consumption frequency (data not shown).

Age, BMI, family history of osteoporosis, smoking habits, and prevalence of fractures were the same in the subgroup with DXA and the subgroup without DXA, both for the athletes and the Tromso controls. None of these variables deviated between the 12 women who completed DXA scan as part of this study and the 12 women who had done DXA scan prior to the study.

## Discussion

In this study older, female athletes had lower BMI, reported higher levels of physical activity, and had a higher prevalence of low BMD, but not of fractures, than women in the general population. Both physical inactivity and a low BMI are associated with osteoporosis and an increased risk of fractures [6, 20–22]. Our findings indicate that the increased risk of having low BMD in long-term, old, female athletes might be due to differences in BMI.

Despite the athletes` increased risk of having low BMD, they did not report more fractures. This might be attributed to a higher bone quality or explained by better muscle strength and function, and improved balance due to high physical fitness, resulting in fewer falls. Cross-country skiing is known to be a sport dependent on cardio-respiratory endurance capacity and balance, but with less load on the skeleton and joints than running [23].

The differences in age, educational level, and use of nutritional- and hormone supplements would favor higher BMD in the athletes and potentially deflate rather than exaggerate the findings in our study.

The most important limitation of this study is the small number of participants in the athlete group, which limits the ability to adjust analyses. The control population lived further north in Norway than the athletes and previous studies have indicated geographical differences with lower BMD in the southern regions in Norway [24]. Hip fractures are also shown to be more prevalent in the south [25], supporting an impact of a possible fracture-protective variable in the athletes, independent of BMD. Fractures could be due to high-energy trauma (and therefore not osteoporotic fragility fractures) and were not adjudicated (i.e. verified by medical personnel/x-ray). Ideally all athletes and controls should have been measured on the same DXA device, and by the same investigator.

## Conclusion

In this pilot study older, female, long-term cross-country skiers seem to be at risk of having low BMD, but not at increased risk of fractures compared to less physically active controls. The negative association between BMD and long-term, high levels of PA might be associated with a lower BMI in the athletes. Because of this, and the numerous other beneficial effects of being active in old age, we do not have evidence to discourage old female athletes from continuing with their sport. The relationship between endurance exercise, BMI, osteoporosis and fracture risk in women and men should be explored in a larger study.

## Abbreviations

aOR: adjusted odds ratio
BIAS: Birkebeiner Ageing Study
BMD: Bone Mineral Density
BMI: Body Mass Index
DXA: Dual-energy X-ray Absorptiometry
OR: Odds ratio
PA: Physical Activity
SD: Standard deviation
SPSS: Statistical Package for the Social Sciences
T-score: The number of SD above or below the mean BMD for a healthy 30-year-old adult of the same sex and ethnicity
